# Unique human orbital morphology compared with that of apes

**DOI:** 10.1038/srep11528

**Published:** 2015-06-25

**Authors:** Eric Denion, Martin Hitier, Vincent Guyader, Audrey-Emmanuelle Dugué, Frédéric Mouriaux

**Affiliations:** 1 Inserm, U 1075 COMETE, Avenue de la côte de nacre, Caen, 5 Avenue de la côte de nacre, 14033 Caen cedex 9, France; 2 Department of Ophthalmology, CHU de Caen, Avenue de la côte de nacre, 14033 Caen cedex 9, France, 14032 Caen cedex 5, France; 3 Medical School, Unicaen, pôle des formations des recherches en santé, 2 rue des Rochambelles, CS 14032, 14032 Caen cedex, France; 4 Department of Otolaryngology - Head & Neck Surgery CHU de Caen, Avenue de la côte de nacre 14033 Caen cedex 9, France; 5 Department of Anatomy, pôle des formations des recherches en santé, 2 rue des Rochambelles, CS 14032, 14032 Caen cedex, France; 6 SASU ThinkR, 63 rue de la Pigacière, 14000 Caen, France; 7 Department of statistics, Centre François Baclesse, 3 avenue du Général Harris, 14000 Caen, France; 8 Department of Ophthalmology, CHU Pontchaillou, 2 rue Henri Le Guilloux, 35033 Rennes Cedex 9, France; 9 Université de Rennes 1, 2 rue du Thabor CS 46510, 35065 Rennes cedex, France

## Abstract

Humans’ and apes’ convergent (front-facing) orbits allow a large overlap of monocular visual fields but are considered to limit the lateral visual field extent. However, humans can greatly expand their lateral visual fields using eye motion. This study aimed to assess whether the human orbital morphology was unique compared with that of apes in avoiding lateral visual field obstruction. The orbits of 100 human skulls and 120 ape skulls (30 gibbons; 30 orangutans; 30 gorillas; 30 chimpanzees and bonobos) were analyzed. The orbital width/height ratio was calculated. Two orbital angles representing orbital convergence and rearward position of the orbital margin respectively were recorded using a protractor and laser levels. Humans have the largest orbital width/height ratio (1.19; p < 0.001). Humans and gibbons have orbits which are significantly less convergent than those of chimpanzees / bonobos, gorillas and orangutans (p < 0.001). These elements suggest a morphology favoring lateral vision in humans. More specifically, the human orbit has a uniquely rearward temporal orbital margin (107.1°; p < 0.001), suitable for avoiding visual obstruction and promoting lateral visual field expansion through eye motion. Such an orbital morphology may have evolved mainly as an adaptation to open-country habitat and bipedal locomotion.

A convergent (forward-facing) orbit, a feature of predators, is marked in animals such as owls, hawks, felids and primates^1^,^2^,^3^. In mammals, convergent orbits ensure good stereoscopic vision through overlapping monocular visual fields, but limit the extent of lateral visual fields, while divergent (lateral-facing) orbits allow panoramic visual fields with little overlap between monocular fields^1^,^2^,^3^,^4^,^5^,^6^. In primates, including humans, the orbital margin is considered to be flat and therefore inscribed in a plane^1^,^4^,^7^,^8^. Hence Cartmill’s^1^ authoritative definition^4^,^5^,^7^,^9^ of orbital convergence as “the dihedral angle between the mid-sagittal plane and the plane of the orbital margin”.

Humans and their closest relatives, the non-human apes^10^,^11^,^12^, form the Hominoidea superfamily^11^. With some of the most convergent orbits among mammals^4^,^7^,^9^, these anthropoid primates are assumed to have a limited lateral visual field^4^,^5^,^6^,^13^. However, we have demonstrated^14^,^15^ that “base visual field” (BVF, i.e. visual field with the eyes facing straight ahead) is much less extensive than “eye-motion visual field” (EMVF, i.e. visual field with allowed eye motion) in humans^15^. Therefore, among mammals, humans may be unique in combining overlapping monocular visual fields with large lateral visual fields, through eye abduction^15^. Among primates, humans have the largest width/height ratio of the palpebral fissure, and this is believed to promote EMVF extension^16^,^17^. Nothing is known about EMVF and its underlying anatomic basics in other primates. On examining a few skulls, we noted that the human orbital margin seemed to be set the furthest back of all members of the Hominoidea superfamily. We hypothesized that the rearward human orbital margin may minimize lateral visual obstruction and increase EMVF. In the present study, we compared human and ape orbital morphologies to assess whether the human orbital margin was exceptionally elongated and whether orbital convergence and the lateral orbital margin positions were different in humans and non-human apes.

## Results

### Orbital width, height and width/height ratio

#### Orbital W/H ratio

**Figure 1:**
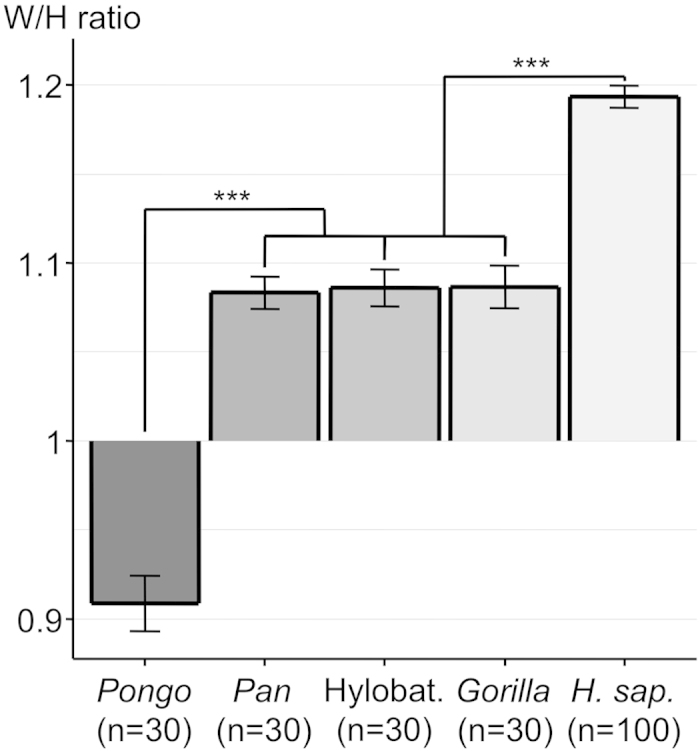
Histogram of orbital width/height (W/H) ratio (mean +/− SEM) of the different genera. ***p < 0.001. Abbreviations used: Hylobat. = Hylobatidae; H. Sap. = *Homo sapiens*.

**Figure 2:**
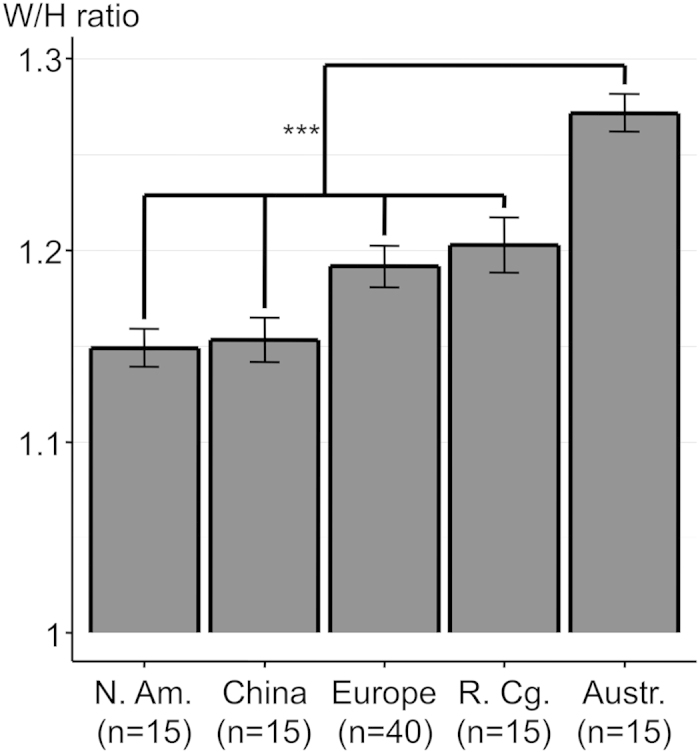
Histogram of the orbital width/height (W/H) ratio (mean + /– SEM) in different human populations. ***p < 0.001. Abbreviations used: N. Am. = North America; R. Cg. = Republic of the Congo; Austr. = Australia.

To assess whether the elongation of the human orbital margin was as exceptional as that of the overlying palpebral fissure, we calculated the orbital width/height (W/H) ratio. Humans have by far the largest orbital W/H ratio, significantly higher (p < 0.001) than that of the group comprised of *Gorilla*, Hylobatidae and *Pan* (Fig. 1, Table 1). This group has a significantly larger W/H ratio (p < 0.001) than that of *Pongo*. We also wanted to know if there was any W/H ratio difference among the different human sub-populations (Native Americans, Chinese, Europeans, Congolese, Aboriginal Australians). The highest ratio was found in Aboriginal Australians (1.27) and was significantly different (p < 0.001) from that of the group comprised of all the other populations (Fig. 2).

#### Orbital CA, OA and OA-CA values

**Figure 3:**
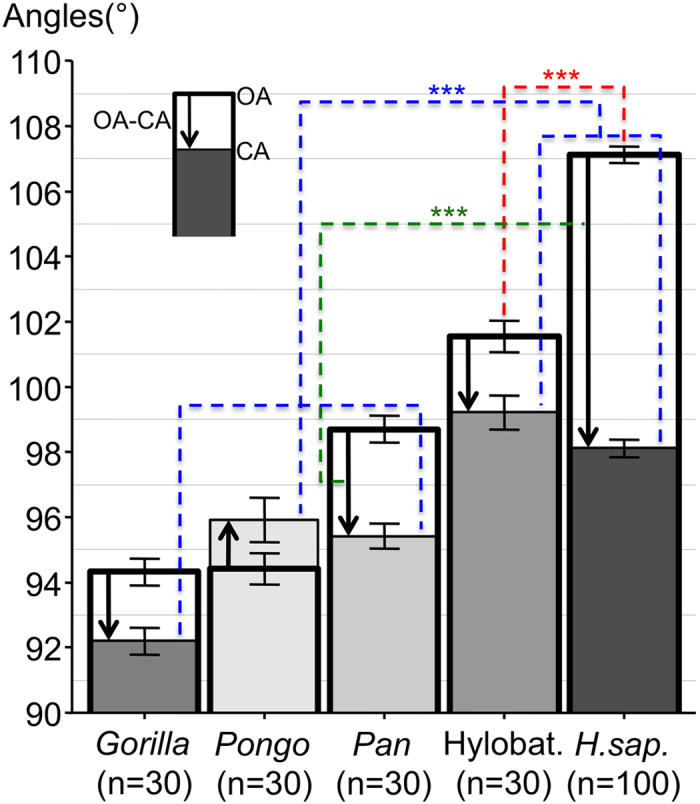
Histogram of orbital angle values (mean + /– SEM) of the different genera. Columns filled with different grey-levels and limited by a thin line denote the convergence angle (CA). White columns limited by a thick line denote the opening angle (OA). The difference between OA and CA is represented by an arrow. The arrow points up in all genera except *Pongo* in whom CA < OA. ***p < 0.001. Abbreviations used: Hylobat. = Hylobatidae; *H. Sap.* = *Homo sapiens*.

The convergence angle (CA) and opening angle (OA) respectively reflected how convergent the orbit was and how rearward the temporal orbital margin position was. The difference between OA and CA (OA-CA) was calculated to study the discrepancy between CA and OA. The group comprised of Hylobatidae (CA = 99.2°) and humans (CA = 98.1°) had significantly (p < 0.001) less convergent orbits than the group comprised of *Gorilla, Pan* and *Pongo* (Fig. 3, Table 1). In humans, we measured a 107.1° OA and calculated a 9° OA-CA. Both of these values were significantly higher than those of the non-human apes (p < 0.001) (Fig. 3, Table 1).

**Figure 4:**
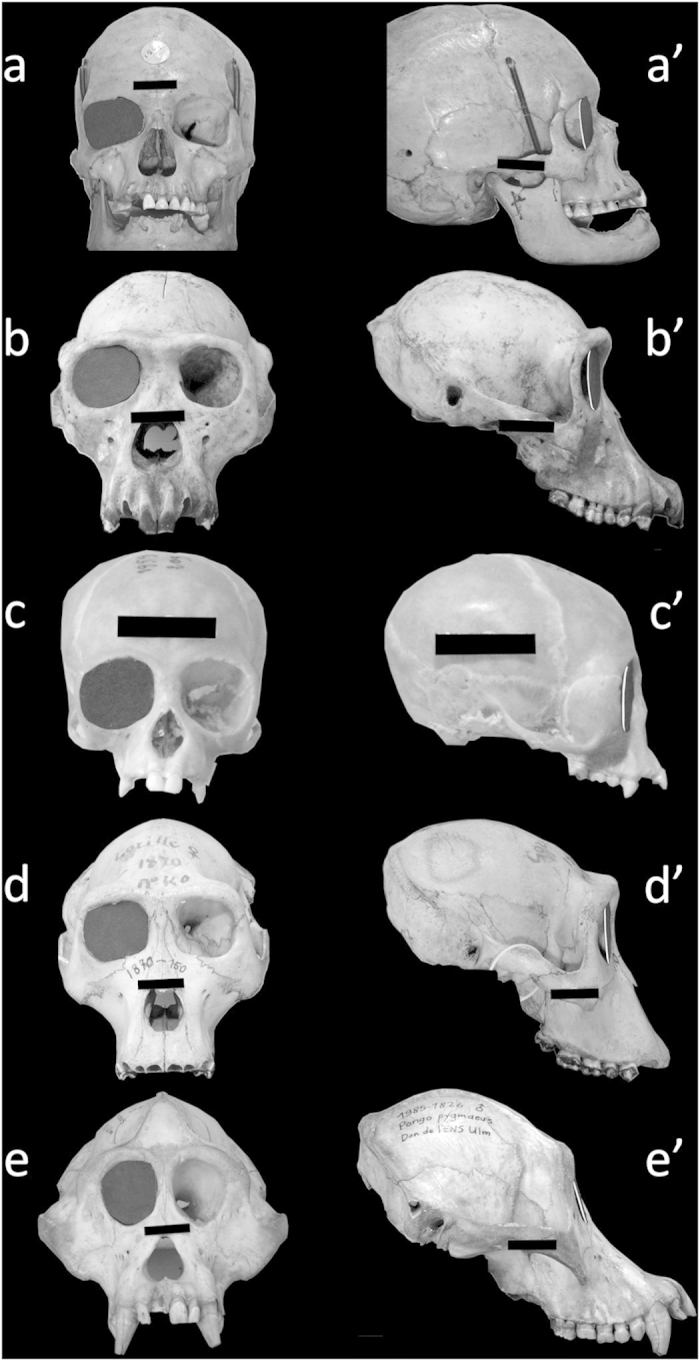
The human orbital margin falls short of being inscribed in a plane. Front and side views of human and ape skulls. A 3-cm wide black landmark affixed to each skull gives the scale. Specimen a/a’ = *Homo sapiens*; specimen b/b’ = *Pan troglodytes*; specimen c/c’ = *Hylobates muelleri*; specimen d/d’ = *Gorilla gorilla*; specimen e/e’ = *Pongo pygmaeus*. A black cardboard lid (representing the convergence angle) has been placed in the right orbital plane of each skull. In humans, the lateral orbital margin is far more rearward than the lid (a’) because of the high discrepancy between convergence and opening angles. In non-human apes, this discrepancy is lower and the cap is much closer to the lateral orbital margin (b’,c’,d’,e’). The specimens are from the Natural History National Museum (Paris, France) anthropological collections (a/a’) and Comparative Anatomy Laboratory (b/b’ to e/e’).

A zero OA-CA value would denote no discrepancy between OA and CA and would imply that the orbital margin is flat and therefore inscribed in a plane. The highest OA-CA value recorded in humans denoted a high discrepancy between CA and OA and showed that the human orbital margin fell short of being inscribed in a plane. As illustrated in Fig. 4, a flat lid cannot therefore close the human orbital margin but comes closer to closing the orbital margins of non-human apes who have a lower OA-CA.

The orbital W/H ratio and OA-CA were supposed to promote lateral visual field extension. We therefore wanted to know if these factors were correlated. A positive correlation was found between the orbital W/H ratio and orbital OA-CA values (0.64 [0.58; 0.69]: p < 0.001).

**Figure 5:**
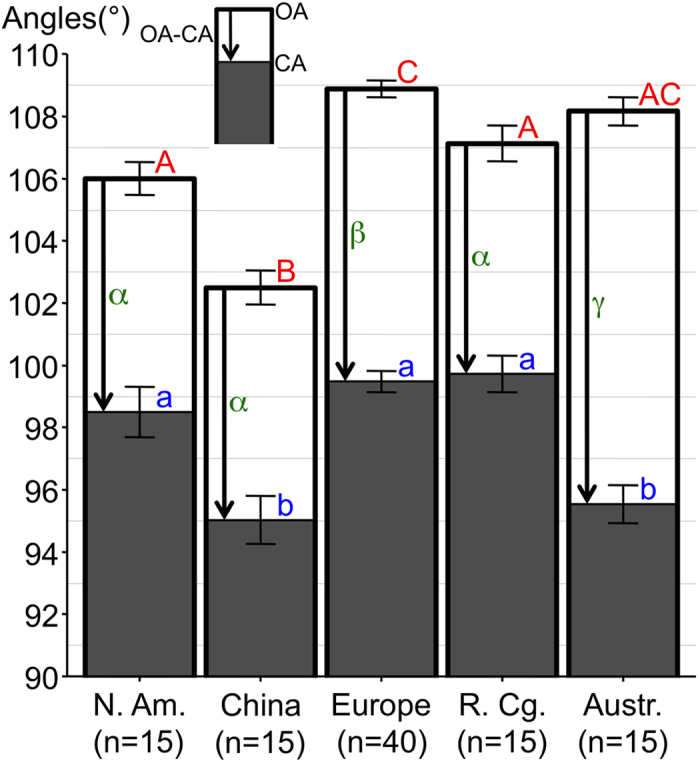
Histogram of orbital angle values (mean + /– SEM) in different modern human populations. The columns filled with dark-grey and limited by a thin line correspond to the convergence angle (CA). White columns limited by a thick line correspond to the opening angle (OA). The difference between OA and CA (OA-CA) is represented by an arrow. Statistically significant differences (p < 0.05) for OA, CA and OA-CA among the human populations were noted using red upper case letters, blue lower case letters and green Greek letters respectively. For the OA, there are significant differences between group A (North America and Republic of the Congo) and group C (Europe); group A and group B (China); group AC (Australia) and group B. For the CA, there are significant differences between group a (North America, Europe and Republic of the Congo) and group b (China and Australia). For the OA-CA, there are significant differences between group α (North America, China and Republic of the Congo) and group β (Europe); group α and group γ (Australia); group β and γ. Abbreviations used: N. Am. = North America; R. Cg. = Republic of the Congo; Austr. = Australia.

For the different human populations (Fig. 5), the OA of skulls from Europe was significantly greater (p values range: <2 × 10^−16^ to 0.012) than that of any other population except Australians. The OA of skulls from China was significantly lower (p values range: <2 × 10^−16^ to <9.2 × 10^−6^) than that of any population. Moreover, the CA of the populations group comprised of skulls from the Republic of the Congo, Europe and North America was significantly higher (p values range: 1.9 × 10^−7^ to 0.0012) than that of the populations group comprised of skulls from Australia and China. The OA-CA was significantly greater (p values range: 3.8 × 10^−14^ to 1.1 × 10^−8^) in skulls from Australia than in any other population.

### Gender influence on evaluation of the measured parameters

No sexual dimorphism was noted for the CA. Female OAs were, on average, 0.4° larger than male OAs. This difference was less than our measurement error (see Methods) and was therefore considered to be insignificant. For the W/H ratio, in humans, male ratios were, on average, 0.04 larger than those of females. This difference was greater than our measurement error and was therefore considered to be significant (p < 0.001). Because the human sex ratio is 1.31 (Table 2), the higher W/H found in human males could potentially bias the result indicating that the human W/H ratio is significantly larger than that of non-human apes. However, the multi-way analysis of variance followed by Tukey’s range test showed that the higher W/H ratio found in human males did not change the fact that the human W/H ratio was significantly larger (p < 0.001) than that of non-human apes.

## Discussion

The human palpebral fissure width/height (W/H) ratio is highest among primates^16^,^17^. Besides the width of the palpebral fissure, the underlying orbital morphology could greatly influence the “eye-motion visual field” (EMVF^14^,^15^). The convergent human orbit, assumed to provide limited lateral visual field extent^4^,^5^,^6^,^13^, in fact allows for significant lateral visual field expansion through eye abduction^14^,^15^. The comparison of human and ape orbital morphology was undertaken to appreciate factors likely to promote or limit EMVF. To the best of our knowledge, our study is the largest comparative series analyzing human and non-human apes orbits^4^,^7^ (Table 3) to date. However, one of the limitations of this study was the difficulty in obtaining a balanced sex ratio. Female skulls were especially difficult to find for gorillas.

As originally recommended by Broca himself^18^, we used the neuro-ocular plane (NOP) as a reference plane to orient skulls and take all the angular measurements in order to ensure effective and precise comparisons between modern humans and other primates^19^,^20^. We used the exact same anatomical landmarks as Broca to materialize the NOP i.e. on both sides the center of the optic foramina and a point equidistant from the superior and inferior orbital margins^18^. To quote Cartmill^1^ “Visual convergence is not a simple univariate quantity. The orientation of the apparatus of vision can be estimated by several different procedures non two of which yield uniformly comparable results.” To measure the convergence angle (CA) we used purposedly the NOP as a reference plane and not, as originally recommended by Cartmill a reference plane perpendicular to both the mid-sagittal plane and to the orbital plane. In humans and apes, the visual pathways and the NOP are transverse, and perpendicular the body axis^19^. Hence, in the orbit, the NOP provides a meridional cut through the globe and through the horizontal recti muscles^19^. It can therefore be considered, as Strait and Ross^21^ did for the horizontal recti muscle, that the NOP is approximately perpendicular to the orbital plane. Moreover, the NOP is perpendicular to the mid-sagittal plane. As a reference plane to record the CA, the NOP is therefore close to the reference plane (i.e. perpendicular to both the mid-sagittal plane and to the orbital plane) initially defined by Cartmill^1^. Besides, as discussed later, using the NOP as a reference plane allows meaningful discussion regarding eye motion influence on visual field eccentricity and regarding published anatomical about eyeball position into the orbit in humans and apes.

To record convergence angle (CA) values, which vary in the same direction as those of temporal visual field eccentricity, we measured the CA between the orbital plane (OP) and the part of the sagittal plane which is anterior rather than posterior to the orbital margin, as had been done in two previously published studies^4^,^7^. Our study yielded CA values close to previously published ones^4^,^7^ (Table 3) with the advantage of having data from many more skulls. The opening angle (OA) was assessed to study how rearward the lateral orbital margin position was. In 1939 Winckler reported measurements of the “inclination of orbit entry relative to the frontal plane” in modern human skulls. This measurement, whose closest equivalent in our study is OA, indicated in his words “the degree to which the outer orbital rim is more rearward than the inner orbital rim”^22^. However he gave no indications about the skulls’ orientation relative to the frontal plane whereas the skulls were oriented using the NOP as a reference plane in our study. The human orbital base has been described as flat with its margin inscribed in a plane^1^,^4^,^8^. The high discrepancy between OA and CA, which is unique to humans and denoted by OA-CA, proves that these notions are erroneous. Hence, far from being flat, the human orbital margin is warped so that its outer part is much more rearward than if it was simply a continuation of the inner part. In Fig. 4 (side view), this complex three-dimensional curvature accounts for the pronounced forward concavity of the human lateral orbital margin^23^,^24^.

The term “frontal” does not describe properly orbital orientation because if often conflates orbit convergence and orbit frontation^4^. Frontation is, to quote Cartmill, “a measure of the extent to which the two orbits face forward toward the end of the snout (as in ape or man) rather than upward toward the skull roof (as in a crocodile)”. Humans and non-human apes have therefore similarly high frontation^1^. Furthermore, frontation is not expected to be correlated with a horizontal parameter like the maximum horizontal extent of overlap of the two monocular visual fields^4^. Our study also deals with a horizontal parameter, i.e. with the influence of orbital morphology on horizontal visual field in humans and non-humans apes. We therefore believe, as previously recommended^4^, that reporting orbital convergence but not frontation is acceptable in our study.

The issue of orbital proportions has traditionally been approached with Broca’s orbital index, that is “(Height of orbit × 100) / Width of orbit”^23^,^24^,^25^. In humans, this index yields figures lower than 100, which conveys the idea that the human orbit is shorter (less tall) than it is wide. In this study on the contrary, the orbital W/H ratio yielded figures higher than 1 in humans, which conveys the idea that the human orbit is wider than it is high. This approach makes it easy to parallel Kobayashi and Kohshima’s finding that modern humans have the largest W/H palpebral fissure ratio of all primates^16^,^17^. The elongated human palpebral fissure, believed to allow visual field expansion through ample eye movement^16^,^17^ appears to have an underlying orbital margin whose unique morphology serves the same purpose. Interestingly, we found that the orbital W/H ratio was significantly higher in Aboriginal Australians skulls than in all the other human sub-groups as reported by Winckler^22^.

In accordance with previously published reports^4^,^7^, we found that human and Hylobatidae orbits were significantly less frontal (higher CA) than those of the group comprised of *Gorilla*, *Pongo* and *Pan* (p < 0.001). This finding, combined with the fact that the human orbit has the largest W/H ratio compared with that of non-human apes, suggests a unique morphology favoring lateral vision. More specifically, while convergent, the human orbit has by far the largest OA and OA-CA values (p < 0.001), reflecting the fact that the lateral orbital margin is far more rearward in humans than in non-human apes (Fig. 4). This finding is not obvious from frontal orbital views. This must account for our finding that human orbital morphology differs from that of non-human apes, whereas a general closeness of *Pan* and human orbits using Fourier analysis of the orbital margin based on frontal photographs was reported^25^. Using his aforementioned system, Winckler reported an “inclination of the orbit entry relative to the frontal plane”, with values between 15 and 17.5°^22^. These values, that would be 105° and 107.5° relative to the sagittal plane, are close to those recorded in our study (mean: 107.1° ± 0.245).

In the NOP, an average 65.7% of the human eyeball length is located in front of the line joining both lateral orbital margins (external bicanthal line)^26^. For this reason, plainly relating eye volume to orbit volume in humans (32% for Schultz^27^) leads to an overestimation of the orbit volume fraction the eye really occupies (20% for Bron *et al.*^28^). In non-human apes, the eyeball is much less protruding than in human. In one *Hylobates lar* (gibbon) and one *Pan troglodytes* (chimpanzee), the eyeball portion located in front of the external bicanthal line using computed tomography cross-sections in the NOP was 11.11%^29^ and 34.78%^30^ respectively. Schultz has reported that, in chimpanzees, the eye lies deep in the orbit which protects the eye amply on all sides and that in orangutans the eyeball reaches slightly beyond the orbital margin^27^. Any photograph of a living gibbon or gorilla^31^,^32^,^33^ shows that their eyeball is deeply tucked into their orbits as well. The human eyeball position in the orbit, much further forward than that of non-human apes, must increase the efficiency of the human lateral orbital margin, which is much more rearward than that of non-human apes, in avoiding lateral visual obstruction, especially when the eyeball is moved outward^15^. This point is relevant, Land having reported that the maximum excursion of eyes in humans and monkeys is similar (+/−45°)^34^. It should be mentioned that a significant (p = 0.013) positive correlation between the forward position of the human eyeball in the orbit and the degree of visual field gained through eye motion in the temporal quadrant has been found^15^. In this study, overall, the orbital W/H ratio and OA-CA values were positively correlated which suggests that orbital horizontal elongation and double orientation serve the same purpose in increasing the extent of the lateral visual field. We have no valid explanations to account for the inter-continental variability of OA, CA and OA-CA observed in our study. We do not relate them simply to global knowledge of anthropometric morphology data, as an increase in nose breadth from north to south, or an increase in bi-zigomatic breadth from west to east^35^.

In everyday life, the eyes rarely remain in the primary position of gaze^14^,^15^,^36^ and we have recently shown that in humans, the “base visual field” (BVF), i.e the visual field with the eyes pointing straight ahead, is much less extensive than the EMVF, i.e. the visual field including eye motion^15^. This is especially true in the temporal quadrant where the median EMVF surface area is 46% more than that of the median BVF. These results are in accordance with reports that humans readily use eyeball rather than head movements to scan their environment^6^,^14^,^15^,^16^,^17^,^36^,^37^. These results also suggest that, as previously hypothesized^15^, humans may be unique among mammals in combining overlapping monocular visual fields and, through eye motion, large (enlarged) lateral visual fields. This issue is tackled in much greater depth in another report (submitted).

The human rearward lateral orbital margin (RLOM) could be a by-product of selection for other aspects of craniofacial anatomy; in other words, an exaptation. Loss of the snout with posterior facial retraction below the anterior cranial fossa in modern humans may be such an aspect^38^. This retraction may have positioned the zygomatic bone, and hence the lateral orbital margin, more posteriorly. However, compared with archaic *Homo* species and the non-human apes, modern humans have a very spherical (globular) cranial vault with a frontal squama that rises steeply above the orbits^39^,^40^ and this may have driven the upper lateral orbital margin forward. Compared with non-human apes, modern humans eat soft, highly processed foods and do not spend much time chewing^38^,^41^. Accordingly, modern humans have masticatory muscles that are much less developed than those of non-human apes^38^. In anthropoid primates, the line of action of the anterior temporalis muscle is roughly vertical^42^. In humans, posterior facial retraction has resulted in a more posteriorly placed anterior temporalis muscle, with a line of action which is expected to have more of an antero-posterior component than is found in non-human apes. However, not much stress transfers to the upper face, including the postorbital septum, during chewing, in anthropoid primates including humans^38^,^43^. Furthermore, assuming that there is more antero-posterior strain on the postorbital septum in humans than in the non-human apes, the expected response would be to add bony mass^38^,^44^ in the zone under strain, the result of which would not be expected to change the position of the lateral orbital margin. Finally, the human anterior temporalis muscle, which is proportionally thinner than that of non-human apes, may provide less support for the postorbital septum. However, the influence of this factor on the position of the human RLOM position is unsubstantiated.

To quote Lieberman^45^ “heads defy many efforts to simplify because they are, by nature, complex and highly integrated systems”. The RLOM likely represents a compromise among many factors including the demands of the temporal fossa content and the demands of the orbit. Apart from exaptation, there are good reasons to think that natural selection has driven the evolution of a RLOM in humans. The human RLOM does not offer much lateral eyeball protection, which may have had little negative selective pressure in humans. Indeed, humans live in a branchless environment with much less risk of branch-related eyeball trauma than the non-human apes who almost exclusively inhabit tropical forests^31^,^32^,^33^,^46^,^47^. Of course, many prosimians (e.g. lemurs) presumably exposed to as much risk of branch-related eyeball trauma as non-human apes, lead a successful arboreal existence without a deep positioning of their eyeballs in their orbits. The point here is not that a protective lateral orbital margin is mandatory for safe arboreal existence but that it may be for the non-human apes, may have been for early hominins and is no longer in the case of modern humans. Apart from eyeball trauma, the human RLOM and anterior eyeball position in the orbit may result in UVB-related eyeball conditions like pterygium or cataracts developed in the nasal aspect of the crystalline lens^48^,^49^. However, these conditions are usually non-blinding^48^. Hence, far from being a “design fault” in the human visual system^48^, the RLOM position and anterior eyeball position in the orbit may represent a trade-off between usually non-blinding UVB-related eyeball conditions and a large visual field^48^, enlarged through eye motion^15^,^16^,^37^, which may aid survival^48^. Humans are ground-dwellers^31^, live in more open spaces than tropical forests^41^,^50^,^51^ and, being the only habitual mammalian bipeds^31^,^50^,^52^,^53^, have most of their visual targets at or parallel to ground level. Compared to knuckle-walking, human bipedal locomotion involves a higher head position and a more forward-facing orbital plane orientation relative to the frontal plane^21^. This overlooking, forward-facing orbital position comes in handy in humans whose large, heavy heads are – according to Galileo’s principle of similitude^17^,^54^,^55^ – much more difficult to move than those of smaller primates. In primates, the eye scales with greater negative allometry with respect to body mass than the orbit does^27^,^56^,^57^. The eyes of large primates (e.g. humans) therefore fill proportionally less orbital volume that the eyes of small primates^56^,^57^. Large primates therefore have proportionally more orbital space for oculomotor muscles^17^. Owing to Galileo’s principle of similitude^17^,^54^,^55^, this favors swift and ample eyeball movements in large primates, especially humans^6^,^14^,^16^,^36^,^58^,^59^. Set in their overlooking anatomical position, the human eyes may thus efficiently scan their environment, mostly at or parallel to ground level. This process, very useful in challenging environments^37^, saves head movements, increases spatial awareness and vigilance through visual and visual field exploration, the RLOM avoiding obstruction of the EMVF^14^,^15^.

## Methods

### Study material

A total of 220 skulls (440 orbits) were studied at the Natural History National Museum and its Comparative Anatomy Laboratory (Paris, France), at the Museum of Natural Sciences (Brussels, Belgium) and the Royal Museum for Central Africa (Tervuren, Belgium). These included 30 Hylobatidae (gibbons) skulls, 30 *Pongo* (orangutan) skulls, 30 *Gorilla* skulls, 30 *Pan* (chimpanzees and bonobos) skulls and 100 *Homo sapiens* (human) skulls (Table 2).

### Orbital width, height and width/height ratio

For both orbits of each skull, maximum orbit width and height were recorded using a thin, metal ruler (model “1051.02”, Facom, New Britain, CT, USA) or calipers (model “815A”, Facom, New Britain, CT, USA) when, owing to rounded orbital margins, orbital points were out of reach of the flat ruler. The orbital width/height (W/H) ratio was then calculated.

### Orbital angles definitions

The Neuro-Ocular Plane (NOP) is the plane that, in the primary gaze position (looking straight ahead into the distance), contains the center of both crystalline lenses, optic discs, and optic foramina^19^,^20^. In the primary gaze position, the pupil is equidistant from the superior and inferior orbital margins^18^,^28^. We therefore defined the NOP, as Paul Broca did in 1873^18^, as the plane which runs symmetrically through both optic foramina and through a point located mid-way between the highest and lowest points of the orbital margin.

Orbital convergence was evaluated with the convergence angle (CA), which is commonly used for that purpose^1^,^4^,^5^,^7^,^9^,^13^. The more or less rearward position of the temporal orbital margin was evaluated by the opening angle (OA). The CA and OA were measured in the NOP, which allows reproducible head orientation in space, and therefore can be used for reliable comparison between humans and other species^20^.

**Figure 6:**
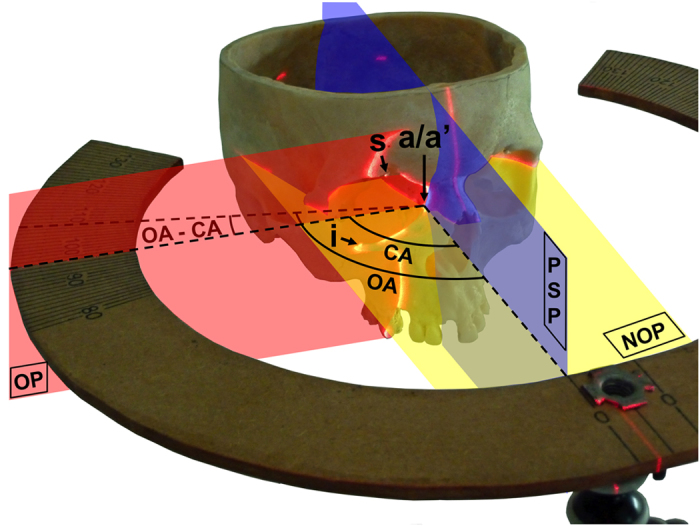
Overview of the convergence angle and opening angle. The sagittal plane and the neuro-ocular plane (NOP: yellow) are represented by a cross-laser. The protractor is positioned in the NOP. The most anterior point of the orbital margin is denoted by *a*. The point where the NOP crosses the inner orbital margin is denoted by *a’*. In this skull, points *a* and *a’* coincide. The highest and lowest orbital points are denoted by *s* and *i* respectively. The orbital plane (OP) crosses points *i*, *s* and *a*. Its position is shown using a red line for laser crossing points *i*, *s* and *a*. The para-sagittal plane (PSP) is parallel to the sagittal plane and crosses points *a* and *a’*. The CA is the angle between OP and PSP, measured using the protractor. The OA is the maximum angle allowing point *a’* to be illuminated by the line laser placed postero-laterally in the NOP. The difference between the CA and the OA is denoted by “OA-CA”.

CA measurement requires the placement of 3 orbital margin points which together define the orbital plane (OP): the highest, lowest and foremost points^1^, respectively denoted by *s*, *i* and *a*. The CA is then defined as the angle – measured in the NOP – between the OP and the midsagittal plane (Fig. 6).

OA measurement requires the placement of point a’ defined as the intersection of the NOP and the inner orbital margin. The OA is then defined as the widest angle allowing a view of a’ when looking postero-laterally at the orbit in the NOP (Fig. 6).

### Orbital angles measurement method

A cross-laser (reference “253003”, Magnusson, Longpont sur Orge, France) mounted on a flexible camera tripod (GorillaPod SLR zoom, Joby, San Francisco, USA) was used to materialize the sagittal plane and the NOP. A wooden protractor prototype was mounted on a flexible camera tripod (GorillaPod SLR, Joby, San Francisco, USA), allowing proper positioning of the protractor in the NOP.

The position of point *a* was determined using a line laser (reference “GPLL5”, Bosch, USA) mounted on a camera tripod (reference “AK-2”, Vanguard, Canton, China). Using a set square, the line laser was placed perpendicular to both the NOP and the sagittal plane. Keeping this orientation constant, the line laser was displaced antero-posteriorly until it crossed the inner orbital margin. The crossing point was denoted by *a* (Fig. 6). The OP was materialized using the line laser mounted on its camera tripod and crossing points i, s and a. In some skulls, bony interference with the line laser made proper alignment of these points impossible. In such cases, the OP position was determined by trial and error, aligning two of the three points and using the thin metal ruler placed in the plane of the line laser to reach the third point. In Fig. 6, points a and a’ share the same location. When this was not the case, the protractor was centered on point *a*, in a plane parallel to the NOP, to record the CA. The lower the CA value, the more convergent the orbital margin orientation.

OA measurement required the use of the line laser mounted on its camera tripod and placed perpendicular to both the NOP and the sagittal plane. Keeping this orientation constant, the line laser, whose source was placed in the NOP, was displaced gradually in a postero-lateral position. The most postero-lateral position allowing point *a’* illumination defined the opening angle. The higher the OA, the more rearward the temporal orbital margin position.

### Statistical analysis

Statistical analysis was performed using R (http://www.r-project.org/). Orbital W/H ratio, OA, CA and OA-CA values were compared using a Student test and with correction of the type I error using the Holm–Bonferroni method. Pairwise comparisons between each pair of taxa (humans, *Gorilla*, Hylobatidae, *Pan*, *Pongo*) and between each of the different human populations (from Africa, Australia, China, Europe and North America) were conducted. To be considered significant, the observed values differences had to be strictly superior to the measurement error (see below) and have a p-value < 0.05.

Pearson’s product-moment correlation was used to assess whether orbital W/H and OA-CA values were positively correlated, for all genera. This correlation yields a coefficient with a value varying between −1 and 1. A zero coefficient value indicates no correlation. The higher or lower the coefficient value, the higher the positive or negative correlation.

Differences between right and left measurements on OA, CA and W/H ratio were measured using a Student test for paired values. For CA, the difference (mean [95% confidence interval]) was 0.47° [0.04 : 0.9]; For OA, the difference was 0.35° [0.05 : 0.76]; For orbital width the difference was 0.04 mm [0 : 0.16]; For orbital height, the difference was 0.14 mm [0.02 : 0.26]. For all these parameters, the upper limit of the 95% confidence interval was below our measurement error. We therefore chose to consider that the right and left measurements were the equivalent of one value measured twice.

Gender influences on OA, CA and the W/H ratio were measured using multi-way analysis of variance. Tukey’s range test was used as a post hoc test to compare *Gorilla*, *Homo*, Hylobatidae, *Pan* and *Pongo* with one another (using pairwise comparisons between each pair of taxa).

### Measurement error evaluation

Twenty skulls (2 females skulls and 2 males skulls, i.e. a total of 4 skulls for Hylobatidae, *Pongo*, *Gorilla*, *Pan* and *Homo*) were chosen at random. For each skull, the CA and OA of both orbits that had already been measured were measured a second time 11 to 25 months later. We called *m1* the value measured the first time; *m2* the value measured the second time and *m3* the average of *m1* and *m2*. We calculated the absolute value of the difference between *m3* and *m1*, for each of the 40 measurements. The measurement error was estimated by taking the upper value of the 95% confidence interval. The same principle was applied to angle difference (AO-AC), orbital width, orbital height and orbital width/height ratio. Measurement errors were 1.33° degree for OA, 0.93° for OA; 1.12° for OA-CA; 0.44 mm for orbital width; 0.35 mm for orbital height; 0.015 for the orbital width/height ratio.

## Additional Information

**How to cite this article**: Denion, E. *et al.* Unique human orbital morphology compared with that of apes. *Sci. Rep.* **5**, 11528; doi: 10.1038/srep11528 (2015).

## Figures and Tables

**Table 1:** Summary of the results recorded in this study.

Skulls	*Gorilla*	*Pongo*	*Pan*	Hylobatidae	Human
	30	30	30	30	100
OA	94.3° ± 0.401	94.4° ± 0.489	98.7° ± 0.408	101.6° ± 0.486	107.1° ± 0.245
CA	92.2° ± 0.406	95.9° ± 0.680	95.4° ± 0.388	99.2° ± 0.522	98.1° ± 0.282
OA-CA	2.1° ± 0.294	−1.5° ± 0.534	3.3° ± 0.249	2.3° ± 0.241	9.0° ± 0.208
W/H ratio	1.087 ± 0.012	0.909 ± 0.016	1.083 ± 0.009	1.086 ± 0.010	1.193 ± 0.006

Abbreviations used: OA = opening angle; CA = convergence angle; OA-CA = difference between OA and CA; W/H ratio = orbital Width/Height ratio. Figures are given as mean + /– SEM.

**Table 2:** Information about the 220 skulls included in this study.

FAMILY, *Genera*	*Genera* and *species*	n	M	F	UG	SR
HYLOBATIDAE	/	30	15	14	1	1.07
*Hylobates*	*H. muelleri*	8	5	3	0	
	*H. lar*	7	3	4	0	
	*H. agilis*	2	0	1	1	
	*H. molloch*	2	1	1	0	
	*H. pileatus*	2	1	1	0	
*Nomascus*	*N. concolor*	2	1	1	0	
	*N. gabriellae*	2	1	1	0	
	*N. leucogenys*	2	1	1	0	
	*N. siki*	1	1	0	0	
*Symphalangus*	*S. syndactylus*	2	1	1	0	
HOMINIDAE	/	190	94	70	26	1.34
*Gorilla*	*/*	30	21	9	0	2.33
	*G. gorilla*	21	16	5	0	
	*G. beringei*	7	5	2	0	
	*G.*; unknown species	2	0	2	0	
*Pan*	/	30	11	16	3	0.69
	*P. troglodytes*	18	5	10	3	
	*P. paniscus*	12	6	6	0	
*Pongo*	*/*	30	15	9	0	1.66
	*P. pygmaeus*	28	14	8	6	
	*P. abelii*	2	1	1	0	
*Homo*	/	100	47	36	17	1.31
	*H. sapiens* (Europe)	40	20	20	0	
	*H. sapiens* (Australia*)	15	10	5	0	
	*H. sapiens* (China)	15	5	4	6	
	*H. sapiens* / (N. Am.**)	15	3	1	11	
	*H. sapiens* / (Rp. Congo)	15	9	6	0	

Abbreviation used: n = number. Footnotes: * =Aboriginal Australians skulls; **Native Americans skulls. Abbreviations used: n = number; M = Male; F = Female; UG = Unknown Gender; SR = sex ratio; *H. = Hylobates*; *N.* = *Nomascus*; S. = *Symphalangus*; *G. = Gorilla*; *P.* = *Pan* or *Pongo*; *H.* = *Homo*; Rp. Congo = Republic of the Congo.; N. Am. = North America.

**Table 3:** Comparison of convergence angle values recorded in this study with published ones.

	Present study		Published studies		
	CA*	Number of skulls	CA** / eq. CA***	Number of skulls	References
*Gorilla*	92.2° ± 0.406	30	80.4° ± 5.320 / 99.6°	5	Ross CF, 1995
*Pan*	95.4° ± 0.388	30	80.6° ± 3.262 / 99.4°	6	Ross CF, 1995
*Pongo*	95.9° ± 0.680	30	82° ± 1.581 / 98°	4	Ross CF, 1995
Human	98.1° ± 0.282	100	79.3° / 100.7°	2	Heesy CP, 2004
Hylobat.	99.2° ± 0.522	30	73.5° ± 3.507 to 77.6° ± 4.641 / 102.4° to 106.5°	18	Ross CF, 1995

Abbreviations used: CA = convergence angle, eq. = equivalent, Hylobat. = Hylobatidae Footnotes: *Figures are given as mean +/– SEM. **Figures are given as mean +/− SD. ***The equivalent CA value (°) is “180 – CA” and allows meaningful comparison to be made between the published CA values and the CA values recorded in this study (see Discussion for details).

## References

[b1] Cartmill M. Arboreal adaptations and the origin of the order primates. In The Functional and Evolutionary Biology of Primates (ed. Tuttle R.) 97–122 (Aldine. Atherton, Chicago, 1972).

[b2] Cartmill M. Rethinking primate origins. Science 184, 436–443 (1974). 4819676 10.1126/science.184.4135.436

[b3] Lieberman D. E. Holding up and moving the head. In The Evolution of the Human Head (ed. Lieberman D. E.) 338–373 (The Belknap Press of Harvard University Press, Cambridge, Massachusetts, 2011).

[b4] Heesy C. P. On the relationship between orbit orientation and binocular visual field overlap in mammals. Anat. Rec. A Discov. Mol. Cell. Evol. Biol. 281, 1104–1110 (2004). 15470671 10.1002/ar.a.20116

[b5] Heesy C. P. Ecomorphology of orbit orientation and the adaptive significance of binocular vision in primates and other mammals. Brain Behav. Evol. 71, 54–67 (2008). 17878718 10.1159/000108621

[b6] Ankel-Simons F. Eyes and eyesight. In Primate Anatomy. An Introduction. Third Edition (ed. Ankel-Simons F.) 444–476 (Elsevier, Amsterdam, 2007).

[b7] Ross C. F. Allometric and functionnal influences on primate orbit orientation and the origins of the Anthropoidea. J. Hum. Evol. 29, 201–227 (1995).

[b8] Rouvière H. Organes des sens. In Anatomie Humaine Descriptive et Topographique. Tome I. Tête, Cou et Tronc (ed. Rouvière H.) 272–373 (Masson, Paris, 1924).

[b9] Ross C. Adaptative explanation for the origins of the anthropoidea (primates). Am. J. Primatol. 40, 205–230 (1996). 10.1002/(SICI)1098-2345(1996)40:3<205::AID-AJP1>3.0.CO;2-1 31918518

[b10] Müller S., Hollatz M. & Wienberg J. Chromosomal phylogeny and evolution of gibbons (Hylobatidae). Hum. Genet. 113, 493–501 (2003). 14569461 10.1007/s00439-003-0997-2

[b11] Groves C. P. Order primates. In Mammal Species of the World: a Taxonomic and Geographic Reference. Third Edition (eds. Wilson D. E. & Reeder D. M.) 111–184 (The Johns Hopkins University Press, Baltimore, 2005).

[b12] Prado-Martinez J. *et al.* Great ape genetic diversity and population history. Nature 499, 471–475 (2013). 23823723 10.1038/nature12228 PMC3822165

[b13] Lieberman D. E. Sense and sensitivity: vision, hearing, olfaction, and taste. In The Evolution of the Human Head (ed. Lieberman D. E.) 374–413 (The Belknap Press of Harvard University Press, Cambridge, Massachusetts, 2011).

[b14] Denion E., Dugué A. E., Augy S., Coffin-Pichonnet S. & Mouriaux F. Sunglasses with thick temples and frame constrict temporal visual field. Optom. Vis. Sci. 90, 1450–1455 (2013). 24141635 10.1097/OPX.0000000000000081

[b15] Denion E., Dugué A. E., Coffin-Pichonnet S., Augy S. & Mouriaux F. Eye motion increases temporal visual field. Acta Ophthalmol 92, e200–206 (2014). 23586899 10.1111/aos.12106

[b16] Kobayashi H. & Kohshima S. Unique morphology of the human eye. Nature 387, 767–768 (1997). 9194557 10.1038/42842

[b17] Kobayashi H. & Kohshima S. Unique morphology of the human eye and its adaptive meaning: comparative studies on external morphology of the primate eye. J. Hum. Evol. 40, 419–435 (2001). 11322803 10.1006/jhev.2001.0468

[b18] Broca P. Sur le plan horizontal de la tête et sur la méthode trigonométrique. Bull. Soc. Anthrop. Paris 2ème série, Tome 8, 48–96 (1873).

[b19] Cabanis E. A. *et al.* CT scanning in the ‘neuro-ocular plane’: The optic pathways as a ‘new’ cephalic plane. Neuro-Ophthalmol. 1, 237–251 (1981).

[b20] Cabanis E. A., Iba-Zizen M. T., Bleynie D. & Coin J. L. Anatomie référentielle et spatiale des voies visuelles: P.N.O. et P.N.O.T.O. In L’imagerie en Ophtalmologie (eds. Cabanis E. A., Bourgeois H. & Iba-Zizen M. T.) 325–336 (Masson, Paris, 1996).

[b21] Strait D. S. & Ross C. F. Kinematic Data on Primate Head and Neck Posture: Implications for the Evolution of Basicranial Flexion and an Evaluation of Registration Planes Used in Paleoanthropology. Am. J. Phys. Anthropol. 108, 205–222 (1999). 9988382 10.1002/(SICI)1096-8644(199902)108:2<205::AID-AJPA6>3.0.CO;2-F

[b22] Winckler G. Anatomie et histologie de l’orbite et des annexes du globe oculaire. In Traité d’Ophtalmologie. Tome Premier (eds Bailliart P., Coutela C., Redslob E., Velter E. & Onfray R.) 204–330 (Masson, Paris, 1939).

[b23] Saraux A., Lemasson C., Offret H. & Renard G. Chapitre premier. L’orbite. In Anatomie et Histologie de l’Oeil. Deuxième Edition (eds Saraux A., Lemasson C., Offret H. & Renard G.) 3–18 (Masson, Paris, 1982).

[b24] Bron A. J., Tripathi R. C. & Tripathi B. J. The bony orbit and paranasal sinuses. In Wolff’s Anatomy of the Eye and Orbit. Eighth Edition (eds. Bron A. J., Tripathi R. C. & Tripathi B. J.) 1–29 (Chapman & Hall medical, London, 1997).

[b25] Schmittbuhl M., Le Minor J. M., Allenbach B. & Schaaf A. Shape of the orbital opening: individual characterization and analysis of variability in modern humans, Gorilla gorilla, and Pan troglodytes. Ann. Anat. 181, 299–307 (1999). 10.1016/s0940-9602(99)80049-1 10363113

[b26] Cabanis E. A. *et al.* Anatomie biométrique et quantitative oculo-orbitaire et encéphalique. In L’imagerie en Ophtalmologie (eds. Cabanis E. A., Bourgeois H. & Iba-Zizen M. T.) 336–364 (Masson, Paris, 1996).

[b27] Schultz A. H. The size of the orbit and of the eye in primates. Am. J. Phys. Anthropol. 26, 389–408 (1940).

[b28] Bron A. J., Tripathi R. C. & Tripathi B. J. 1997 The eyeball and its dimensions. In Wolff’s Anatomy of the Eye and Orbit. Eighth Edition (eds. Bron A. J., Tripathi R. C. & Tripathi B. J.) 211–232 (Chapman & Hall medical, London, 1997).

[b29] Saban R. *et al.* Tomodensitométrie *in vivo* chez *Hylobates lar lar* L. 1771 (Catarhinii, Anthropomorpha), dans le plan neuro-oculaire (P.N.O.). C. R. Acad. Sci. 299, 151–156 (1984).

[b30] Saban R. *et al.* Tomodensitométrie *in vivo* du Chimpanzé (*Pan troglodytes*, Catarhinii, Anthropomorpha) dans le plan neuro-oculaire (P.N.O.). C. R. Acad. Sci. 300, 341–346 (1985). 3922581

[b31] Rowe N. Apes. In The Pictorial Guide to the Living Primates (ed. Rowe N.) 206–233 (Pogonias Press, Charlestown, 1996).

[b32] Nowak R. M. Gibbons, or Lesser Apes. In Walker’s Primates of the World (ed. Nowak R. M.) 168–173 (The Johns Hopkins University Press, Baltimore, 1999).

[b33] Nowak R. M. Great Apes. In Walker’s Primates of the World (ed. Nowak R. M.) 173–186 (The Johns Hopkins University Press, Baltimore, 1999).

[b34] Land M. F. Eye movements of vertebrates and their relation to eye form and function. J Comp Physiol A. 201, 195–214 (2015). 10.1007/s00359-014-0964-5 25398576

[b35] Froment A. La différentiation morphologique de l’homme moderne: congruence entre forme du crâne et répartition géographique du peuplement. C. R. Acad. Sci. 315, 323–329 (1992). 1490226

[b36] Le Grand Y. Chapitre 13: L’orbite et ses muscles. In Optique Physiologique. Tome Troisième: l’Espace Visuel. (ed. Le Grand Y.) 28–41 (Editions de la “Revue d’Optique”,: Paris, , 1956).

[b37] Hedblom E. E. Snowscape eye protection. Development of a sunglass for useful vision with comfort from antarctic snowblindness, glare, and calorophthalgia. Arch. Environ. Health 2, 685–704 (1961). 13712688 10.1080/00039896.1961.10662927

[b38] Lieberman D. E. You are how you eat: chewing and the head. In The Evolution of the Human Head (ed. Lieberman D. E.) 224–280 (The Belknap Press of Harvard University Press, Cambridge, Massachusetts, 2011).

[b39] Lieberman D. E. The evolution of the Head in H. Sapiens. In The Evolution of the Human Head (ed. Lieberman D. E.) 527–603 (The Belknap Press of Harvard University Press, Cambridge, Massachusetts, 2011).

[b40] Lieberman, D. E., McBratney, B. M. & Krovitz, G. The evolution and development of cranial form in *Homo sapiens*. Proc. Natl. Acad. Sci. U S A. 99,1134–1139 (2002). 11805284 10.1073/pnas.022440799 PMC122156

[b41] Lieberman, D. E. The Story of the Human Body (Allen Lane, London, 2013).

[b42] Ross, C. F. & Hylander, W. L. Electromyography of the anterior temporalis and masseter muscles of owl monkeys (Aotus trivirgatus) and the function of the postorbital septum. Am. J. Phys. Anthropol. 112, 455–468 (2000). 10918124 10.1002/1096-8644(200008)112:4<455::AID-AJPA4>3.0.CO;2-4

[b43] Nakashige, M., Smith, A. L. & Strait, D. S. Biomechanics of the macaque postorbital septum investigated using finite element analysis: implications for anthropoid evolution. J. Anat. 218, 142–150 (2011). 21070237 10.1111/j.1469-7580.2010.01316.x PMC3039786

[b44] Ravosa M. J., Noble, V. E., Hylander, W. L., Johnson, K. R. & Kowalski, E. M. Masticatory stress, orbital orientation and the evolution of the primate postorbital bar. J Hum Evol. 38, 667–693 (2000). 10799259 10.1006/jhev.1999.0380

[b45] Lieberman D. E. Final thoughts and speculation. In The Evolution of the Human Head (ed. Lieberman D. E.) 604–613 (The Belknap Press of Harvard University Press, Cambridge, Massachusetts, 2011).

[b46] Sorenson J. Ape (Reaktion Books Ltd., London, 2009).

[b47] Matthews, L. H. *et al.* Le Monde Etrange et Fascinant des Animaux. Deuxième Edition (Sélection du Reader’s Digest, Paris, 1972).

[b48] Coroneo M. T. Albedo concentration in the anterior eye: a phenomenon that locates some solar diseases. Ophthalmic Surg. 21, 60–66 (1990). 2325997

[b49] Coroneo M. T. Pterygium as an early indicator of ultraviolet insolation: a hypothesis. Br J Ophthalmol. 77, 734–739 (1993). 8280691 10.1136/bjo.77.11.734 PMC504636

[b50] Lieberman D. E. Early hominin heads. In The Evolution of the Human Head (ed. Lieberman D. E.) 414–474 (The Belknap Press of Harvard University Press, Cambridge, Massachusetts, 2011).

[b51] Froment A. Evolution humaine et rayonnement solaire. In Le Soleil dans la Peau (eds. Bonnet-Bidaud J. M., Froment A., Moureaux P. & Petit A.) 73–125 (Robert Laffont, Paris, 2012).

[b52] Swindler, D. A. Introduction to the Primates (The University of Washington Press, Washington, 1998).

[b53] Lieberman, D. E. The brain and the skull. In The Evolution of the Human Head (ed. Lieberman D. E.) 182–223 (The Belknap Press of Harvard University Press, Cambridge, Massachusetts, 2011).

[b54] Galileo G. Dialogues Concerning Two New Sciences. Translated by Henry Crew and Alfonso De Salvio (Dover publications Inc., New York, 1954).

[b55] Thompson D. W. On Growth and Form. The complete revised edition (Dover Publications Inc., New York, 1942).

[b56] Kay R. F. & Kirk E. C. Osteological evidence for the evolution of activity pattern and visual acuity in primates. Am. J. Phys. Anthropol. 113, 235–262 (2000). 11002207 10.1002/1096-8644(200010)113:2<235::AID-AJPA7>3.0.CO;2-9

[b57] Kirk E. C. Effects of activity pattern on eye size and orbital aperture size in primates. J. Hum. Evol. 51,159–170 (2006). 16620912 10.1016/j.jhevol.2006.02.004

[b58] Le Grand Y. Chapitre 14: Géométrie des mouvements de l’œil. In Optique Physiologique. Tome Premier: la Dioptrique de l’Oeil et sa Correction (ed. Le Grand Y.) 164–176 (Editions de la “Revue d’Optique”, Paris, 1956).

[b59] Hugonnier R. Physiologie des mouvements oculaires. In Strabismes Hétérophories Paralysies Oculo-Motrices (Les Déséquilibres Oculo-Moteurs en Clinique) (ed. Hugonnier R.) 75–90 (Masson, Paris, 1959).

