# Corrigendum: Unique human orbital morphology compared with that of apes

**DOI:** 10.1038/srep29861

**Published:** 2016-07-20

**Authors:** Eric Denion, Martin Hitier, Vincent Guyader, Audrey-Emmanuelle Dugué, Frédéric Mouriaux

Scientific Reports 5: Article number: 1152810.1038/srep11528; published online: 06252015; updated: 07202016

This Article contains typographical errors.

In the Methods section under subheading ‘Measurement error evaluation’,

“Measurement errors were 1.33° degree for OA, 0.93° for OA; 1.12° for OA-CA; 0.44 mm for orbital width; 0.35 mm for orbital height; 0.015 for the orbital width/height ratio.”

should read:

“Measurement errors were 1.33° degree for CA, 0.93° for OA; 1.12° for OA-CA; 0.44 mm for orbital width; 0.35 mm for orbital height; 0.015 for the orbital width/height ratio.”

